# An on-chip coupled resonator optical waveguide single-photon buffer

**DOI:** 10.1038/ncomms3725

**Published:** 2013-11-12

**Authors:** Hiroki Takesue, Nobuyuki Matsuda, Eiichi Kuramochi, William J. Munro, Masaya Notomi

**Affiliations:** 1 NTT Basic Research Laboratories, NTT Corporation, 3-1 Morinosato Wakamiya, Atsugi, Kanagawa 243-0198, Japan; 2 Nanophotonics Center, NTT Corporation, 3-1 Morinosato Wakamiya, Atsugi, Kanagawa 243-0198, Japan

## Abstract

Integrated quantum optical circuits are now seen as one of the most promising approaches with which to realize single-photon quantum information processing. Many of the core elements for such circuits have been realized, including sources, gates and detectors. However, a significant missing function necessary for photonic quantum information processing on-chip is a buffer, where single photons are stored for a short period of time to facilitate circuit synchronization. Here we report an on-chip single-photon buffer based on coupled resonator optical waveguides (CROW) consisting of 400 high-Q photonic crystal line-defect nanocavities. By using the CROW, a pulsed single photon is successfully buffered for 150 ps with 50-ps tunability while maintaining its non-classical properties. Furthermore, we show that our buffer preserves entanglement by storing and retrieving one photon from a time-bin entangled state. This is a significant step towards an all-optical integrated quantum information processor.

## Abstract (web summary)

Photonic circuits are a promising route to developing scalable quantum technologies, if all the necessary components can be built. Using coupled resonator optical waveguides, Takesue *et al.* present an on-chip single-photon buffer that can delay one photon from a pair for 150 ps while preserving entanglement.

## Graphical abstract

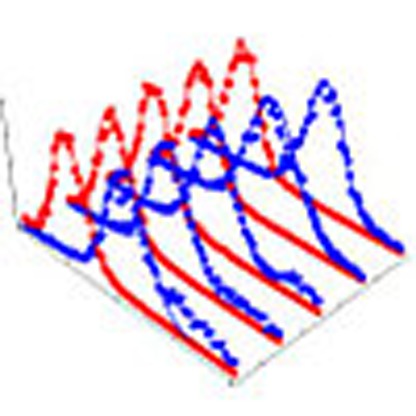

Photonic quantum information processing is now recognized as one of the best ways to achieve large-scale quantum computation^1^ and communication^2^. To date, most photonic quantum information experiments have been performed using bulk optics^1^,^3^. As the size and complexity of these realizations increase, these bulk optics approaches begin to limit and hamper the functionality of the experiments that can be performed. The natural solution is to move to an integrated photonics approach^4^. Such approaches have already shown that on-chip photon sources^5^,^6^,^7^,^8^,^9^,^10^,^11^,^12^, detectors^13^,^14^ and circuit-based gates can be achieved. Non-trivial circuits have been implemented ranging from basic observations of quantum interference^4^,^15^ and multi-photon entanglement manipulation^16^, to the sophisticated implementation of quantum computation tasks such as Shor’s algorithm^17^, quantum walks^18^,^19^ and boson sampling^20^,^21^,^22^,^23^. There is, however, a critical missing element that has yet to be realized on-chip: a quantum buffer. Figure 1a is a schematic of an all-on-chip quantum processor expected in the near future, where quantum functional circuits are integrated with quantum buffers together with photon sources and detectors. A buffer is critical for synchronizing the photons in such an integrated circuit, which becomes much more difficult as the integration and compactness of circuit increases. The buffer can also be used to unsynchronize (temporally stagger) photons so they do not interfere in certain parts of the circuits. Furthermore, a tunable buffer together with other active elements such as optical switches^24^ enables us to realize a ‘quantum field-programmable gate array’, namely a fully programmable integrated quantum optical circuit.

A natural way to realize an integrated single-photon buffer is to use the slow-light effect in optical waveguides^25^. Among several waveguide-based slow-light devices reported so far^26^,^27^, a coupled resonator optical waveguides (CROW) is a promising candidate due to its large bandwidth with small group velocity dispersion^28^,^29^,^30^,^31^,^32^,^33^. A CROW is a one-dimensional array of identical optical cavities, where adjacent cavities are coupled to each other. An extended mode along the waveguide can be formed using a nearest-neighbour interaction. Consequently, a CROW exhibits a transmission bandwidth that is far larger than the individual cavities, whereas the group velocity is significantly reduced inside the band^28^.

In this Article, we demonstrate an integrated single-photon buffer based on a CROW fabricated using silicon photonic crystal (PhC) technologies^32^. With this CROW, whose length is only 840 μm, a pulsed photon from a correlated photon pair source is buffered by up to 150 ps with a tunability of 50 ps, while preserving its quantum correlation with the other photon. Furthermore, we confirm that entanglement could be preserved after storing and retrieving one photon from a time-bin entangled state.

## Results

### CROW based on silicon PhC waveguide

**Figure 1:**
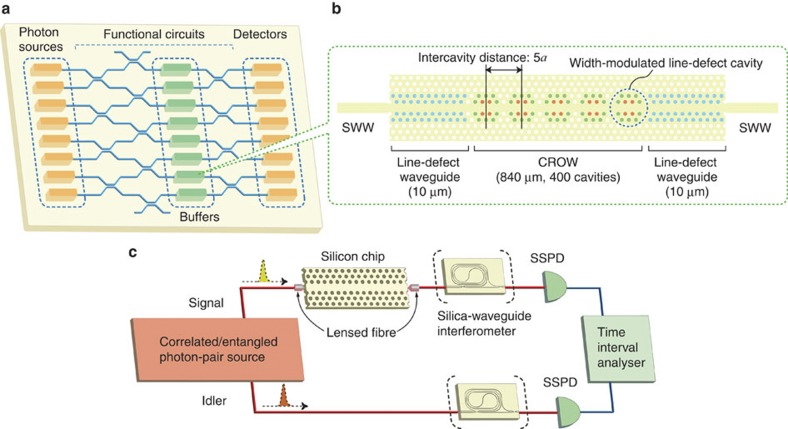
Quantum buffer concept. (**a**) A conceptual diagram of a photonic quantum processor based on integrated quantum photonics including the use of quantum buffers. In principle, such buffers could be placed at any point in the circuit. (**b**) Quantum buffer based on CROW that consists of photonic crystal nanocavities. Silicon wire waveguides (SWW) are integrated with the PhC section to access the CROW. (**c**) Experimental set-up for observing photon buffering and entanglement storage. The silica-waveguide interferometers were inserted in the entanglement storage experiment.

**Figure 2:**
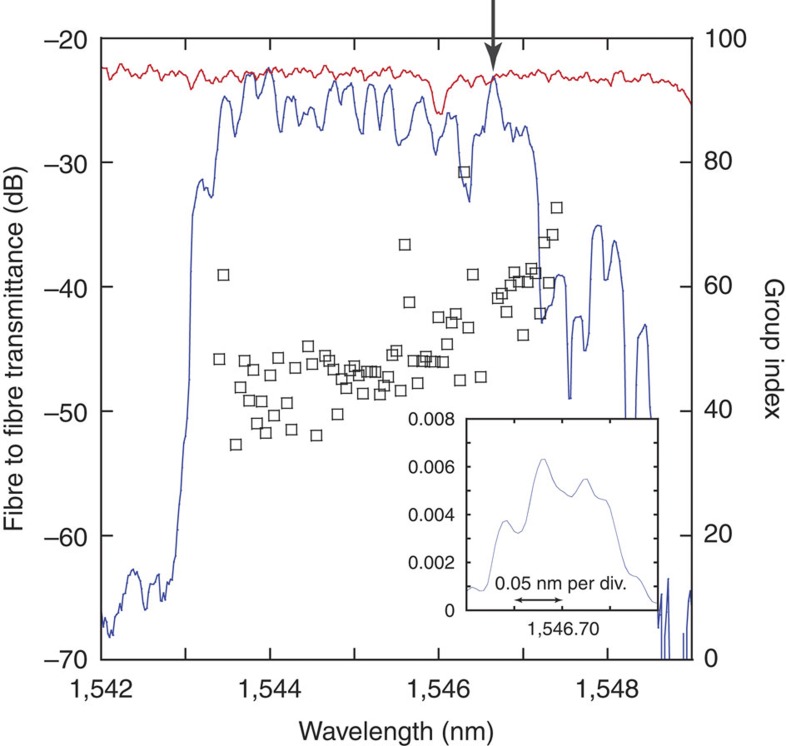
Transmission spectra and group index of CROW at a temperature of 21.6 °C. The blue and red lines show the fibre-to-fibre transmission spectra of the CROW and the reference waveguide, respectively. The resolution bandwidth was 0.2 nm. We chose a 0.2-nm resolution to show the effective loss for the 0.2-nm bandwidth photons used in our experiment. The arrow indicates the signal photon wavelength (1,546.70 nm). The squares denote the group index of the CROW. The inset shows the CROW spectrum around the signal photon wavelength measured with a 0.02-nm resolution bandwidth. The vertical axis corresponds to the fibre-to-fibre transmittance on a linear scale.

Figure 1b shows the design of our CROW^32^, which is based on a width-modulated line-defect cavity in a silicon PhC with a two-dimensional triangular lattice of air holes^34^. The lattice constant *a* of our PhC waveguide is 420 nm, and the intercavity distance is 5*a*. The Q of each cavity is ~10^6^. The number of cavities is 400, which means that the total length is 840 μm. A 10-μm PhC line-defect waveguide is fabricated at each end of the CROW. Figure 2 shows the transmission spectra of the CROW and the reference waveguide at a temperature of 21.6 °C. Here, the transmittance includes the coupling losses between the lensed fibre and the chip for both the input and output facets (~8 dB per point), connector losses (<0.3 dB per point) and the possible extra loss resulting from misalignment of the lensed fibre. The spectra were measured with an amplified spontaneous emission source and an optical spectrum analyser whose resolution bandwidth was set at 0.2 nm. The squares show the group index of the CROW, which was obtained by measuring the propagation time differences of classical pulses in the CROW and the reference waveguide. As observed in Fig. 2, a transmission band was formed between 1,543 and 1,548 nm. Although a peaky structure that was probably caused by fabrication errors was observed^32^, there were several low-loss peaks in the spectrum. In the following experiments, we tuned the chip temperature so that one of the spectral peaks matched the signal photon wavelength of 1,546.70 nm (indicated by the arrow in Fig. 2). The observed temperature dependence of the transmission spectrum was ~0.07 nm °C. The PhC section is integrated with silicon wire waveguides (SWW). We fabricated another waveguide of the same length on the same chip as a reference for the temporal delay, where we replaced the CROW section with a PhC line-defect waveguide. The CROW presented here was not apodized to realize impedance matching between the CROW and the line-defect waveguides. However, the strong intercavity coupling at the intercavity distance of 5*a* moderated the confinement of light inside the CROW, which realized good coupling between the line-defect waveguides and the CROW without apodization, compared with our previous work^32^. Nevertheless, the implementation of apodization is an effective way to smooth the spectral characteristics further.

### Observation of photon buffering

Our experimental set-up for the observation of photon buffering is depicted in Fig. 1c. A photon pair source based on spontaneous four-wave mixing (SFWM) in a dispersion-shifted fibre (DSF) (see Methods for details) generates signal and idler photons. The wavelengths of the signal and idler photons were 1,546.70 and 1,555.53 nm, respectively, with a bandwidth of 0.2 nm. The signal photon was coupled to the silicon chip that included the CROW and the reference waveguide by using lensed fibres, and the photons output from the waveguide were received by a superconducting single-photon detector (SSPD)^35^. The idler photon was directly measured by the second SSPD. The detection signal from the SSPD for the signal (idler) was used as the start (stop) signal for a time interval analyser performing coincidence measurements.

**Figure 3:**
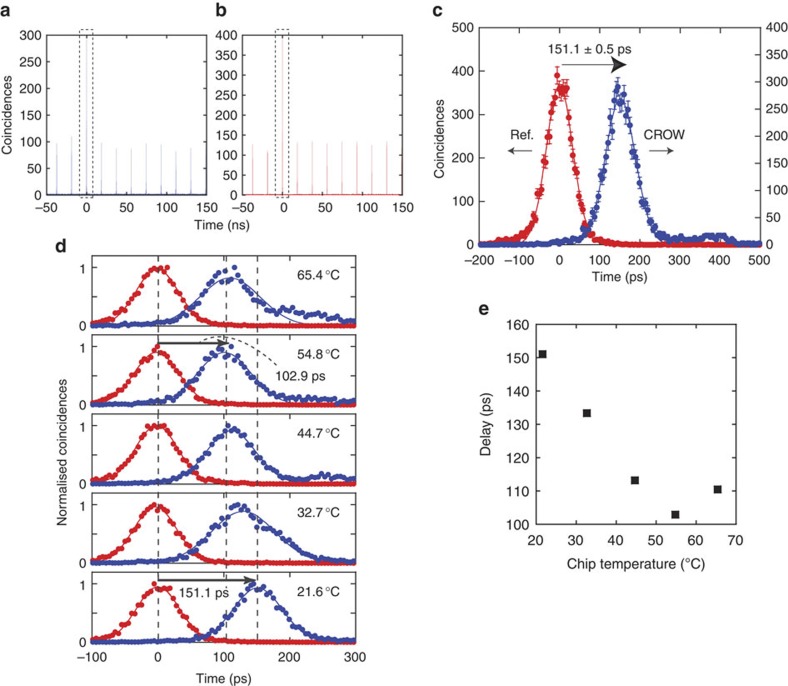
Single-photon buffer experimental results. (**a**,**b**) Time interval histograms when the signal photons were transmitted through (**a**) the CROW and (**b**) the reference waveguide. (**c**) Enlarged image of main peaks observed in dashed boxes in (**a**) and (**b**). The blue and red dots show histograms obtained with signal photons transmitted through the CROW and the reference waveguide, respectively. Error bars were calculated assuming that the number distribution of coincidence counts is Poissonian, and the lines indicate Gaussian fitting to the data. (**d**) Time interval histograms around the main peaks with various chip temperatures. Although the temporal positions of the peaks for the reference waveguide (red dots) remained unchanged, those for the CROW (blue dots) showed strong temperature dependence. The delay as a function of chip temperature is plotted in (**e**). For figs (**a**–**d**), coincidences were counted for 1 million detection events of the signal photons that were used as the start signals for the time interval analyser.

Figure 3 shows time interval histograms of the coincidence counts as a function of the relative delay between the signal and idler detection events. The average signal photon number per pulse was set at 0.13. Fig. 3a,b shows the time interval histograms where the signal photons were transmitted through the CROW and the reference waveguide, respectively. The chip temperature was 21.6 °C. We observed higher peaks at a relative delay near 0 (highlighted by dotted boxes), which correspond to the coincidences caused by the correlated signal and idler photons generated by the same pump pulse. These peaks are significantly higher than other peaks that correspond to accidental coincidences caused by photons generated by different pump pulses, implying the existence of an intensity correlation between the two photons. The cross correlation 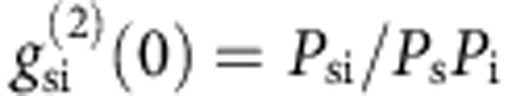, where *P*_si_ is the coincidence detection probability and *P*_s_ (*P*_i_) is the probability of detecting a signal (idler) photon, was measured and found to be 3.25±0.06 for the CROW and 3.10±0.05 for the reference waveguide. When we removed the waveguide from the set-up, 
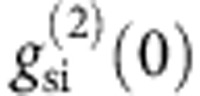
 was 3.22±0.05. Note that 
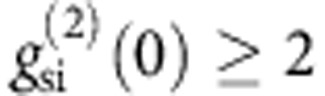
 implies the existence of a non-classical intensity correlation between the signal and idler photons^36^. Thus, our result confirms that the quantum correlation was preserved even after the signal photon had been transmitted through 400 high-Q nanocavities, with virtually no degradation.

The coincidences at the main peaks were regarded as the detection events of quasi-single photons heralded by the detection of the idler photon. Since the detection times of the idler photons were unchanged, we could measure the delay time of the quasi-single photons in the CROW from the temporal shift of the main peaks. Figure 3c shows an enlarged image of the main peaks in Fig. 3a,b, where a clear separation of the two peaks is seen. This result confirmed that the CROW, whose physical length was only 840 μm, stored a single photon for as long as 151.1±0.5 ps. The group index of the reference waveguide was ~5, which means that the speed of the pulsed photon in the CROW was slowed to 1/59 of the light speed in a vacuum. The 1/*e* half-widths of the peaks were 52.7±0.6 ps (CROW) and 46.3±0.4 (reference waveguide). We also observed a slight tail in the coincidence histogram for the CROW. These observations suggest that the small spectral structure of the CROW may have led to the excess broadening of the temporal width and the slight distortion of the pulse shape. Still, the obtained result indicates that our buffer can store a broadband single-photon pulse whose 1/*e* half temporal width is smaller than <30 ps (see Methods).

### Delay tuning

We now demonstrate the tunability of our single-photon buffer. The transmission band of our silicon PhC CROW shifts to a longer wavelength as the chip temperature increases, and the wavelength dependence of an ideal CROW group index forms a sinusoidal curve with its minimum value at the centre of the band^28^ (the experimentally obtained group indices are shown in Fig. 2). This implies that we can shift the dispersion characteristic by changing the temperature, which enables us to tune the buffer time. We varied the chip temperature from 21.6–65.4 °C, while maintaining the wavelength of the signal photon. Here, we set the chip temperature so that the signal photon wavelength matched one of the spectral peaks in the CROW transmission spectrum. The histograms obtained around the main peaks are shown in Fig. 3d which clearly reveal a temperature-dependent delay. We also confirmed the preservation of a non-classical correlation with the idler photons for all chip temperatures. As shown in Fig. 3e, the buffer time was varied from 103–151 ps, suggesting that we realized a tunable buffer with a tuning range of ~50 ps. In free space, such a 50-ps tunability requires a 1.5-cm variable delay compared with 840 μm in our waveguide.

### Storage of entanglement

We have already demonstrated the tunable buffering ability of our CROW. However, if they are to be used in photonic circuits for quantum information tasks, photons buffered by the CROW must preserve quantum coherence with other photons in the circuit. The simplest demonstration of this is to buffer one half of an entangled state and then show that the buffered photon is still entangled after it is released from the CROW. Again using SFWM in a DSF, we generated a high-dimensional time-bin entangled state at a clock frequency of 2 GHz, whose state is given by^37^ (see Methods),


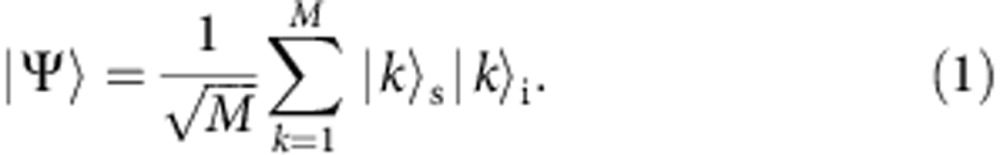


Here, *M* is the number of time slots where the coherence of the pump pulse is preserved and |*k*
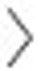
_*x*_ denotes a state where there is a photon at the *k*th time slot in the mode *x* (s: signal, i: idler). As shown in Fig. 1c, the signal photons transmitted through the silicon chip (temperature: 21.6 °C) and idler photons were input into delayed Mach–Zehnder interferometers whose propagation time difference between two arms was 1 ns. These interferometers were made of silica-based optical waveguides fabricated on silicon substrates, and the phase difference between the two arms was tuned by changing the waveguide temperature^38^. The photons from the interferometers were detected by SSPDs, and the coincidences were counted. The coincidence probability *P* is given by (see Methods for details)


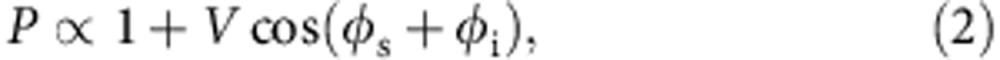


where *V* is the two-photon interference visibility, and *ϕ*_s_ and *ϕ*_i_ denote the phase difference between the two arms of the interferometers for the signal and the idler, respectively.

**Figure 4:**
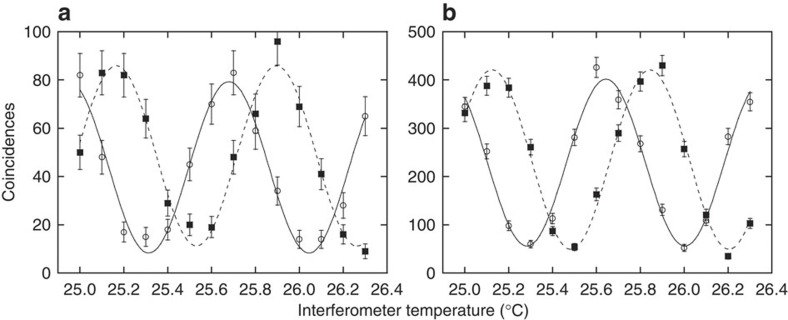
Two-photon interference fringes. (**a**) and (**b**) correspond to fringes with and without the CROW, respectively. Squares and circles show the results when the idler interferometer temperature was tuned to 22.74 and 22.94 °C, respectively. Error bars were calculated assuming that the number distribution of coincidence counts is Poissonian. The visibilities of fitted curves for the fringes shown in (**a**) are 77±5% (22.74 °C) and 81±5% (22.94 °C), which indicates that that the stored entanglement should violate Bell’s inequality. The visibilities for the fringes in (**b**) are 79±3% (22.74 °C) and 76±4% (22.94 °C). The coincidences were counted for (**a**) 500,000 and (**b**) 2 million detection events of the signal photons that were used as the start signals for the time interval analyser.

We measured the coincidences as a function of the signal interferometer temperatures while fixing the idler interferometer temperature at 22.74 and 22.94 °C, which correspond to two non-orthogonal measurements for the idler photons. The two-photon interference measurement is described in detail in Methods. The result is shown by squares (22.74 °C) and circles (22.94 °C) in Fig. 4a, where clear modulations of the coincidence counts can be observed. The data are fitted with equation (2), and the visibilities of the fitted curves were 77±5% (22.74 °C) and 81±5% (22.94 °C). These visibilities suggest that the buffer could preserve high-quality entanglement that violates the Bell’s inequality. We also undertook two-photon interference measurements without the chip, and obtained fringes whose visibilities were 79±3% (22.74 °C) and 76±4% (22.94 °C) (the fringes are shown in Fig. 4b). This result suggests that the degradation of the entanglement visibilities caused by the buffering in the CROW was smaller than the statistical error.

## Discussion

As already stated, the fibre-to-fibre loss of our device was relatively large. The fibre-to-fibre loss consists of the external and internal losses. The external loss includes the coupling loss between a lensed fibre and a chip facet (~8 dB per point), the fibre connector loss (typically <0.3 dB per point), and the loss due to the imperfect alignment of a lensed fibre. The internal loss includes the propagation losses of the CROW, line-defect waveguides and SWWs, and the connection losses between different waveguides. Therefore, it is difficult to isolate the loss of the CROW. However, the transmittance spectra shown in Fig. 2 suggest that some of the CROW transmission peaks have similar losses to that of the reference waveguide, which is expected to be ~2 dB mm^−1^ with the present device. Therefore, we consider that our CROW-based buffer can be introduced with a reasonably small loss into an integrated quantum optical circuit. In addition, it is possible to integrate spot-size converters at both edges of the chip, and thus reduce the coupling loss between the fibres and the chip so that our buffer may find applications in fibre-based quantum information systems. We can achieve a <0.5 dB coupling loss per point, for example with a spot-size converter based on a Si adiabatic taper^39^.

An important figure of merit of a photon buffer is the loss per unit delay. With our CROW at a transmission peak whose loss was the same as that of the reference waveguide (loss ~2 dB mm^−1^), we estimated this value to be 1.1 dB per 100 ps delay, which is comparable to the value for a typical SWW of ~0.8 dB per 100 ps delay (*n*_g_~4.3 and 1.1 dB cm^−1^ loss for transverse electric polarization mode were assumed based on Yamada *et al.*^39^). Moreover, with further improvement of the device fabrication accuracy, we will be able to realize a photon buffer with a smaller loss per unit delay based on the CROW.

In our delay tuning experiment, the peaky structure of the transmission spectrum prevented the continuous tuning of the delay time. The structure was mainly caused by the fluctuation of the centre wavelength and the Q factor of each cavity, and thus can be suppressed as the fabrication accuracy improves. The reflection at the boundaries between the line-defect waveguides and the CROW was another reason for the peaky structure, and this can be eliminated by apodization. Moreover, as described in detail in Methods, the Fabry-Perot oscillation caused by the reflection from each facet of the silicon chip led to a smaller transmission structure that may have resulted in the pulse broadening. We can eliminate this by applying an anti-reflection coating to each facet. With these improvements, we can expect to realize a pulse-broadening-free, continuously tunable single-photon buffer.

Our devices based on a silicon PhC with a two-dimensional triangular lattice of air holes are strongly polarization dependent (transverse electric polarization only). Therefore, our device is a natural candidate for a buffer in quantum information processing circuits based on dual rail or time-bin qubits. Moreover, we can use our CROW-based buffer for a polarization qubit by incorporating it with a polarization diversity technique developed for an SWW^39^.

It is possible to increase the delay time further. One way to achieve this is to increase the length of the CROW at the expense of an increased device footprint. Another way is to increase the group index by decreasing the cavity coupling^28^, which can be realized by increasing the intercavity distance^32^. In this case, we can retain the small device footprint, but the transmission bandwidth is generally reduced. Thus, we can design a CROW-based single-photon buffer flexibly depending on the requirements of a particular quantum information system.

Although the temperature tuning technique demonstrated here is relatively slow (tuning time ~1 ms), it is sufficient for realizing programmable integrated quantum optical circuits where there is no requirement as regards the tuning speed. Nevertheless, a buffer with a faster tuning speed will find more applications for example an active quantum feedforward^40^ on a chip. A possible candidate for a fast tunable buffer is a high-Q PhC cavity dynamically tuned by carrier injection^34^.

The biggest advantage of the CROW-based buffer over other simple delay lines is its compactness: thanks to the large group index achieved in the silicon PhC CROW, our buffer can be more than one order of magnitude smaller than those based on silica or silicon waveguides. In addition to the compactness, the large tunability is another advantage of our buffer. The 50-ps tunability achieved in this experiment corresponds to ~10 mm and ~3.5 mm changes in the lengths of a fibre and an SWW, respectively, which are hard to realize in a compact optical circuit device.

In conclusion, we have demonstrated a single-photon buffer based on a CROW. We showed that a pulsed single photon was buffered for up to 150 ps with 50-ps tunability, and confirmed the preservation of entanglement. We believe that such a quantum buffer will be useful for realizing a flexible and reconfigurable quantum processor on a chip.

## Methods

### Generation of photon pairs using DSF

A pump pulse train with a wavelength of 1,551.1 nm was amplified by an erbium-doped fibre amplifier (EDFA), and then transmitted through optical filters to suppress the amplified spontaneous emission noise generated in the EDFA. The filtered pulse train was launched into a 500 m long DSF. Through the SFWM process in the DSF, two pump photons were annihilated and a correlated photon pair was generated. The generated photons were input into another filter to separate the signal and idler photons, whose wavelengths were 1,546.70 and 1,555.53 nm, respectively, and the spectral width was 0.2 nm for both channels. The signal and idler photons were detected by respective SSPDs, whose detection efficiencies were 14 and 11% for the signal and idler channels, respectively. The dark count rate was ~10 cps for both detectors. The large loss caused by the insertion of the chip meant that the coincidence probability between the two detectors was very small, resulting in a long measurement time. For example, the CROW’s delay measurement whose result is shown by the squares of Fig. 3c took about 30 min. During such a long measurement time, the coupling loss between the fibre and the chip tended to change due to the temperature fluctuation in the laboratory, and led to a fluctuation in the count rate of the SSPD for the signal photons. To suppress the change in the coincidence rate caused by the count rate fluctuation in the signal channel, we used the detection signal from the SSPD for the signal photons as the start signals for the time interval analyser and observed the coincidences for a fixed number of start pulses. As the loss for the idler channel was stable and the contribution of the dark counts to the coincidence measurement was small, thanks to the low-noise characteristics of the SSPD, we were able to count the coincidences (or coincidence probability) stably.

We used different pump pulse sources in photon buffer experiments and entanglement storage experiments. In the photon buffer experiments described above, we used sub-ps pulses with a repetition frequency of 53.65 MHz generated from a fibre mode-locked laser. The pulses were transmitted through an optical filter whose spectral width was 0.2 nm, before being input into the EDFA. The full width at half maximum (FWHM) of the pump pulse was measured to be ~20 ps with an autocorrelator. In the entanglement storage experiments, we used continuous-wave laser light from an external cavity diode laser, which was modulated into 60-ps, 2-GHz frequency pulses using a lithium niobate intensity modulator.

In the entanglement storage experiment, the average signal photon number per pulse was set at 0.01, and the chip temperature was 21.6 °C. The pump coherence time was ~10 μs, which means that *M* in equation (1) was ~2 × 10^4^. We used silica-waveguide interferometers with a 1-ns propagation time difference between two arms, while the temporal interval of our entangled photon pulses was 0.5 ns. This means that a state |*k*
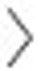
_*x*_ (*x*=s: signal, i: idler) is converted to (|*k*
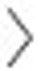
_*x*_+*e*^*iϕ*_*x*_^|*k*+2
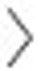
_*x*_)/2, where *ϕ*_*x*_ is the phase difference between the two arms of the interferometer. Then, equation (1) is converted to


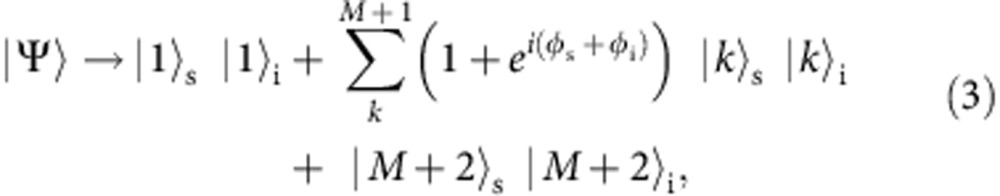


where only the terms that contributed to the coincidence detection are shown and the normalizing term is discarded for simplicity. By ignoring the first and the last terms of the right hand side of the above equation, we obtain the coincidence probability *P* as equation (2). We employed the high-dimensional time-bin entangled state because we can fully use the time domain and thus achieve fast measurement despite the relatively small average photon number per pulse.

### Estimation of photon pulse broadening in CROW

From the *n*_g_ values obtained from the delay tuning experiment and the *n*_g_ curve shown in Fig. 2, the dispersion parameter *β*_2_ was estimated to be ~−3.7 × 10^−20^ s^−2^ m^−1^ at a group index of 59 (where the group velocity dispersion is expected to be maximum in the tuning range). The dispersion length of the CROW for a 20-ps (FWHM) Gaussian pulse calculated using this value was ~4 mm, which is an order of magnitude larger than the device length. This result suggests that the pulse broadening caused by the group velocity dispersion was negligible in our experiment. The 1/*e* half-width of the SSPD jitter, *σ*_sspd_, was measured and found to be 30.4 ps for both channels. Assuming Gaussian jitter and photon pulse broadening, the 1/*e* half-widths of the main peaks of time interval histograms, *σ*_main_, can be expressed as


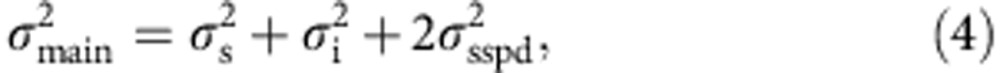


where *σ*_s_ and *σ*_i_ denote the 1/*e* half-widths of the signal and idler photons, respectively. We assume that the idler photon width was the same as the pump pulse width (20 ps FWHM), which means *σ*_i_≃12 ps. Thus, we obtain *σ*_s_≃28 ps for the signal photon width after transmission in the CROW. On the other hand, after the signal photon had been transmitted through the reference waveguide, *σ*_s_ was calculated to be ~12 ps, indicating that the CROW induced a broadening of the signal photon pulse. A possible reason for the broadening is a filtering effect resulting from the fine structure of the CROW transmission spectrum. We measured the spectrum of the peak around 1,546.7 nm using an optical spectrum analyser with a 0.02 nm resolution bandwidth (the inset in Fig. 2), and found that the 3-dB bandwidth of the peak was ~0.12 nm. In addition, the Fabry-Perot oscillation caused by the reflection from each facet of the chip was convoluted with the waveform, leading to a rather complex spectral shape. Although a detailed investigation based on spectral measurement with a better wavelength resolution will be needed, we believe that these small structures in the CROW transmission spectrum resulted in the excess broadening of the photon pulse.

## Author contributions

H.T. and N.M. performed the experiments and data analysis. H.T. and W.J.M. designed the concept of reconfigurable quantum circuits with quantum buffers. M.N. and E.K. designed and fabricated the CROW device. H.T. managed the project. All authors discussed the results and wrote the manuscript.

## Additional information

**How to cite this article:** Takesue, H. *et al.* An on-chip coupled resonator optical waveguide single-photon buffer. *Nat. Commun.* 4:2725 doi: 10.1038/ncomms3725 (2013).
